# Intravitreal dexamethasone implant versus triamcinolone acetonide for macular oedema of central retinal vein occlusion: quantifying efficacy and safety

**DOI:** 10.1186/s40942-018-0114-2

**Published:** 2018-03-26

**Authors:** S. K. Mishra, A. Gupta, S. Patyal, S. Kumar, K. Raji, A. Singh, V. Sharma

**Affiliations:** 1 Department of Ophthalmology Command hospital, Lucknow cantt, 226002 India; 2 Department of Ophthalmology, Army Hospital Research and Referral, Delhi Cantt, New Delhi, 110010 India; 3 Army Hospital Research and Referral, Delhi Cantt, New Delhi, 110010 India

**Keywords:** Central retinal vein occlusion, CRVO, Non ischaemic CRVO, IVTA, Triamcinalone acetonide, Ozurdex, Dexamethasone implant

## Abstract

**Purpose:**

Among the retinal vascular diseases, burden of retinal vein occlusion is most common immediately after diabetic retinopathy. Intravitreal corticosteroids are gaining popularity in managing macular edema (ME) of RVO. Our study compares efficacy and safety of intravitreal triamcinolone (IVTA) and dexamethasone implant (IVD) over 6 months.

**Methods:**

This comparative, prospective, randomized study on 40 patients of non-ischemic central RVO with significant ME (> 330 μm) of < 3 months duration. Study was done at Army Research Hospital between Sep-2012 and May-2014 in accordance to Helsinki Declaration. IVD group (n = 20) received Ozurdex^®^ while IVTA group (n = 20) received triamcinolone-acetonide (4 mg/0.1 ml), followed up at day-1 and weeks 4, 8, 12, 24.

**Results:**

At 6 months, mean improvement in best corrected visual acuity and retinal thickness (CMT) in the IVD group was 0.43 logmar and 323 μm and in IVTA group was 0.49 logmar and 322 μm respectively. Proportion of patients achieving ≥ 15 letters was about 40% in both groups. IOP rise was significantly higher in IVTA group at 12 and 24 weeks. In IVTA group ≥ 10 mmHg IOP rise was seen in 60% of patients, 41.6% among them had > 35 mmHg and 66% needed combination treatment and failed to reach baseline line IOP at 6 months. In IVD group, 5 pts had IOP rise with all being < 26 mmHg and were easily managed with single agent with IOP reaching baseline by 6th month in all pts. Relative risk of IOP rise with IVTA is 2.4 times higher compared to IVD. Cataract progression and cataract surgeries were required at significantly higher rates in IVTA group. In IVTA group, cataract progression was seen in 35% patients, with 71.5% requiring cataract surgery at 6 months. IVD group, 10% patients had cataract progression while none required surgery at 6 months. Relative risk of cataract progression with IVTA is 3.5 times higher compared to IVD.

**Conclusion:**

Intravitreal steroids are effective in managing macular edema of retinal vein occlusion, while newer formulation of sustained release dexamethasone implant is significantly safer than IVTA.

## Introduction and purpose

Among the retinal vascular diseases, burden of retinal vein occlusion is most common immediately after diabetic retinopathy [1]. Central RVO (CRVO) impedes blood supply leading to retinal ischaemia, edema and significant ocular morbidity. Incidence is common in persons over 65 years, while affecting either sexes equally [2–4]. Prevalence of CRVO is reported at 0.1–0.4% [2, 5], with worsening of vision related quality of life [6].

**I**ntravitreal corticosteroids are gaining popularity in managing ME of RVO as its effects span over controlling and limiting angiogenesis, re-establishing retinal fluid homeostasis and reducing edema, additionally having anti-apoptotic and anti-proliferative effects. Advantages of intravitreal delivery include localised/targeted action in the retina with additional higher concentration of the drug with prolonged action. Commonly used intravitreal steroids are triamcinolone-acetonide and 0.7 mg dexamethasone implant. Earlier studies have established both steroids in reducing macular edema with visual acuity improvement in patients of CRVO [7, 8].

Our study aims at comparative evaluation of intravitreal triamcinolone (IVTA) and dexamethasone implant (IVD) over 6 months.

## Methods

Study design is prospective, comparative, randomized study, carried out at Army Hospital Research and Referral ophthalmology clinic between Sep-2012 and May-2014. The study is conducted in accordance with Declaration of Helsinki and approved by Institutional Research Board. Signed informed consents were obtained from all patients before enrolling into study. Forty patients of non-ischemic CRVO with significant ME (> 330 μm) of < 3 months duration were included. Clinical diagnosis was confirmed by FFA and OCT (Spectralis, Cirrus) with significant cystoid ME (CME) as defined by CRVO Study Group [9]. The exclusion criteria’s were existence of other retinal vascular diseases (diabetic retinopathy, age related degeneration), glaucoma, previous treatments for CRVO (intravitreals or laser-photocoagulation), iris neovascularization and > 10 disc retinal ischemia in FFA.

This prospective study is designed to evaluate the efficacy and safety of intravitreal triamcinolone and dexamethasone implant over a study period of 6 months. Intravitreal desamethasone group [IVD group (n = 20)] received 0.7 mg dexamethasone implant (Ozurdex^®^, Allergan) while intravitreal triamcinolone acetonide [IVTA group (n = 20)] received 4 mg/0.1 ml triamcinolone-acetonide (Kenacort^®^, BMS). Study starts from the day of the injection and were followed up for a minimum of 6 months. Follow up evaluations were done on day 1 and week 4, followed by monthly evaluations for a minimum duration of 6 months. Reinjections of intravitreals were allowed in cases of recurrent edema and warranting treatment as judged by investigator. Recurrence of ME was defined as a decrease in VA ≤ 2 lines or increase in intraretinal or subretinal fluid, with ME ≥ 320 μm. Evaluations at baseline and follow-up included best corrected visual acuity (BCVA; Snellen chart at 6 m), slit-lamp examination of anterior segment, intraocular pressure (IOP; Goldmann Tonometer), indirect ophthalmoscopy, FFA and OCT. The primary endpoints of the study were BCVA, CMT on OCT, IOP, and cataract progression.

Statistical analysis was done using Stata90 (College Station, USA). Data is presented as median (min–max) or number (%) as appropriate. Continuous baseline characters were compared between groups using Wilcoxon-Ranksum test and categorical baseline characteristics were compared using Fisher’s exact test. The outcomes variable such as BCVA, IOP, CMT were compared between groups using Wilcoxon Ranksum test and within group using Wilcoxon Signed Ranksum test as sample size is small. Statistical significance was defined as value < 0.05. VA is converted to logmar for statistical analysis.

## Results

Of the 40 patients, 20 (11 men, 9 women) received IVTA and 20 (12 men, 8 women) received IVD for ME secondary to CRVO. The sex distribution was similar between groups (P = 0.502), as was the mean patient age (58.6 ± 10.41 vs. 57.9 ± 8.77 years; P = 0.342). The mean follow-up time is 6 months in both groups. Mean baseline measurements in IVD versus IVTA group are BCVA (logmar) (1.06 ± 0.13 vs. 0.99 ± 0.15), CMT (551 ± 17.16 vs. 547.5 ± 13.79 μm) and IOP (16.7 ± 1.16 vs. 16.1 ± 2.60 mmHg) respectively, and were not statistically significant (Table 1). Arterial hypertension was diagnosed in ten subjects (IVD group-4 pts; IVTA group-6 pts). Seven patients had hyperlipidemia (IVD group-3 pts; IVTA group-4 pts). Eight patients were cigarette smokers in IVD group and nine smokers in IVTA group. Two subjects in IVD group required 2 IVD injections during study period. Four subjects in the IVTA group required 2 injections of IVTA during study period.

**Table 1:** Baseline parameters in patients with CRVO

	Dexamethasone (n = 20)	Triamcinolone acetonide (n = 20)
Sex (M/F)	12/8	9/11
Mean age (years ± SD)	58.6 ± 10.41	57.9 ± 8.77
Smoker (n)	8	9
Hypertension (n)	4	6
Hyperlipidemia (n)	3	4
Follow up (months ± SD)	6	6
BCVA (logMAR ± SD)	1.06 ± 0.13	0.99 ± 0.15
CMT (µm ± SD)	551 ± 17.16	547.5 ± 13.79
IOP (mmHg ± SD)	16.7 ± 1.16	16.1 ± 2.60

*BCVA* best-corrected visual acuity, *CMT* central macular thickness, *IOP* intraocular pressure, *NS* not significant

The mean BCVA improved by 0.43 logmar (P < 0.001) in IVD group and by 0.49 logmar (P < 0.001) in IVTA group at study end (Table 2). However, visual acuity gains between groups were not statistically significant (Table 3). CMT improved by 314 μm in IVD group, while in IVTA group CMT improved by 314.9 μm by 6 months after intravitreal injections. While within groups CMT improvements were statistically significant, between groups the improvement was not statistically valid (P = 0.4244) (Tables 2, 4).

**Table 2:** Comparison of baseline and end-of-follow-up parameters in patients with central retinal vein occlusion

	Dexamethasone (n = 20)			Triamcinolone (n = 20)		
	Baseline	After	P	Baseline	After	P
BCVA (log MAR ± SD)	1.06 ± 0.13	0.637 ± 0.54	0.0179	0.99 ± 0.15	0.508 ± 0.45	0.0142
CMT (µm)	551 ± 17.16	237 ± 20.71	0.0050	547.5 ± 13.79	232.6 ± 18.54	0.0050
IOP (mmHg)	16.7 ± 1.16	17 ± 1.94	0.7083	16.1 ± 2.60	22.2 ± 4.93	0.0076

*BCVA* best-corrected visual acuity, *CMT* central macular thickness, *IOP* intraocular pressure, *NS* not significant

**Table 3:** Comparison of logMAR BCVA between Ozurdex and triamcinolone at each follow up visit

	Ozu (n = 20) logmar BCVA-median	Versus baseline; *P* value	Tri (n = 20) logmar BCVA-median	Versus baseline; P value	Ozu versus Tri P value
Baseline	1.079	–	1.0395	–	0.3920
4 weeks	0.778	0.107*	0.778	0.0107*	0.9071
8 weeks	0.602	0.0352*	0.602	0.0246*	1.0000
12 weeks	0.477	0.0208*	0.477	0.0188*	0.6990
24 weeks	0.477	0.0179*	0.389	0.0142*	0.5828

*Statistically significant

**Table 4:** Comparison of CMT between Ozurdex and triamcinolone at each follow up visit

	Ozu (n = 20) mean CMT-µm	Versus Baseline; P value	Tri (n = 20) mean CMT-µm	Versus baseline; P value	Ozu versus Tri P value
Baseline	555	–	552	–	0.5362
4 weeks	450	0.0050*	402	0.0050*	0.4254
8 weeks	350	0.0050*	330	0.0050*	0.4037
12 weeks	270	0.0049*	240	0.0050*	0.6201
24 weeks	232	0.0050*	230	0.0050*	0.4244

*Statistically significant

At 6 months, rise in IOP was statistically significant only in the IVTA group (Table 2). IOP lowering medication was initiated in 12 eyes (60%) in the IVTA group and in 5 eyes (25%) in IVD group in 6 months. All the 5 eyes in the IVD group had IOP of < 26 mmHg and was readily controlled by single antiglaucoma medication and reached baseline by 6 months. Peak IOP was recorded at second month of follow up. In IVTA group IOP lowering medication was initiated in 12 eyes (60%). 5 eyes (41.6%) had IOP of more than 35 mmHg. Eight eyes (66.6%) needed combination eye drops and did not normalise by 6 months of study. The statistical analysis of IOP comparison between dexamethasone and triamcinolone was significant at 12 and 24 weeks with triamcinolone group showing increased IOP (Table 5). Within the Ozurdex group the change in IOP from baseline was statistically significant up till 12 weeks but by the end of 24 weeks this change became statistically insignificant but in the triamcinolone group this was statistically significant by the end of 6 month period (Table 5). Relative risk of IOP rise with IVTA is 2.4 times higher compared to IVD.

**Table 5:** Comparison of IOP between Ozurdex and triamcinolone at each follow up visit

	Ozu (n = 20) median IOP (mmHg)	Versus baseline; P value	Tri (n = 20) median IOP (mmHg)	Versus baseline; P value	Ozu versus Tri P value
Baseline	17	–	15	–	0.4144
4 weeks	18	0.0113*	23	0.0.0076*	0.2532
8 weeks	19	0.0036*	27	0.0058*	0.1918
12 weeks	18	0.0048*	26	0.0058*	0.0354*
24 weeks	16	0.7083	25	0.0076*	0.0110*

*Statistically significant

**Fig. 1:**
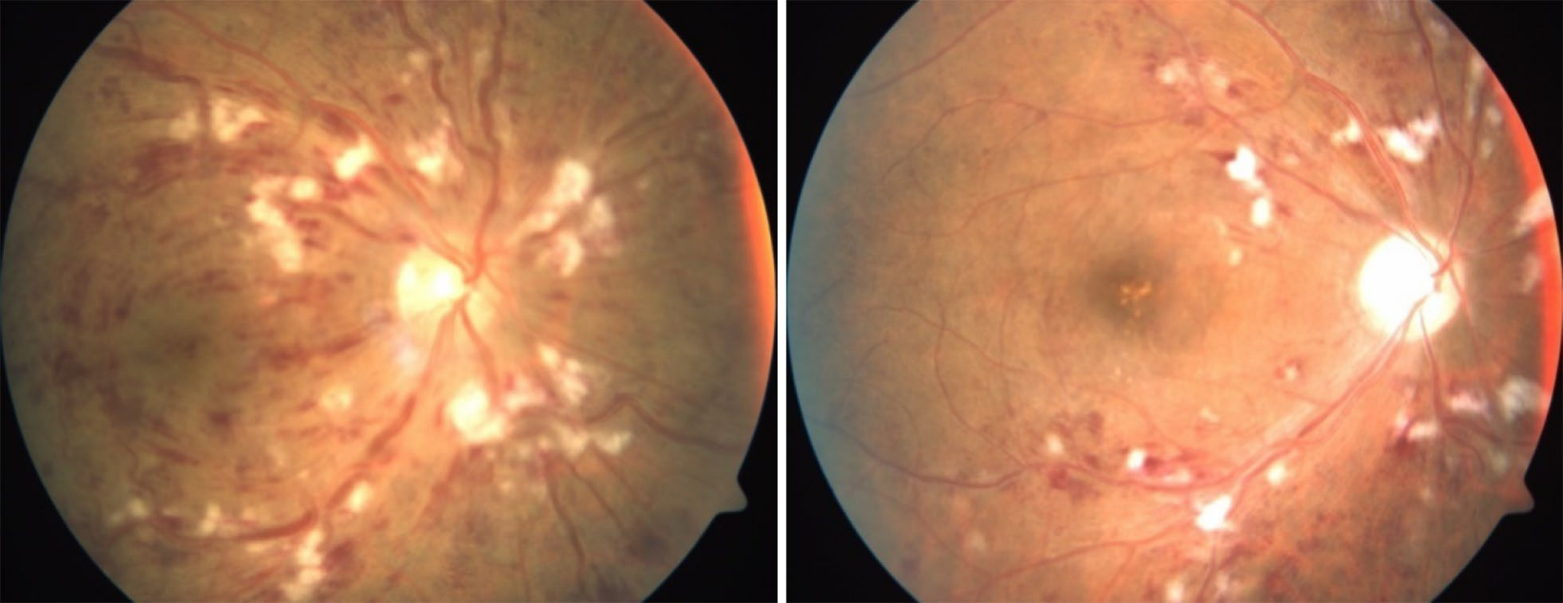
Fundus photo pre and post Inj Ozurdex

**Fig. 2:**
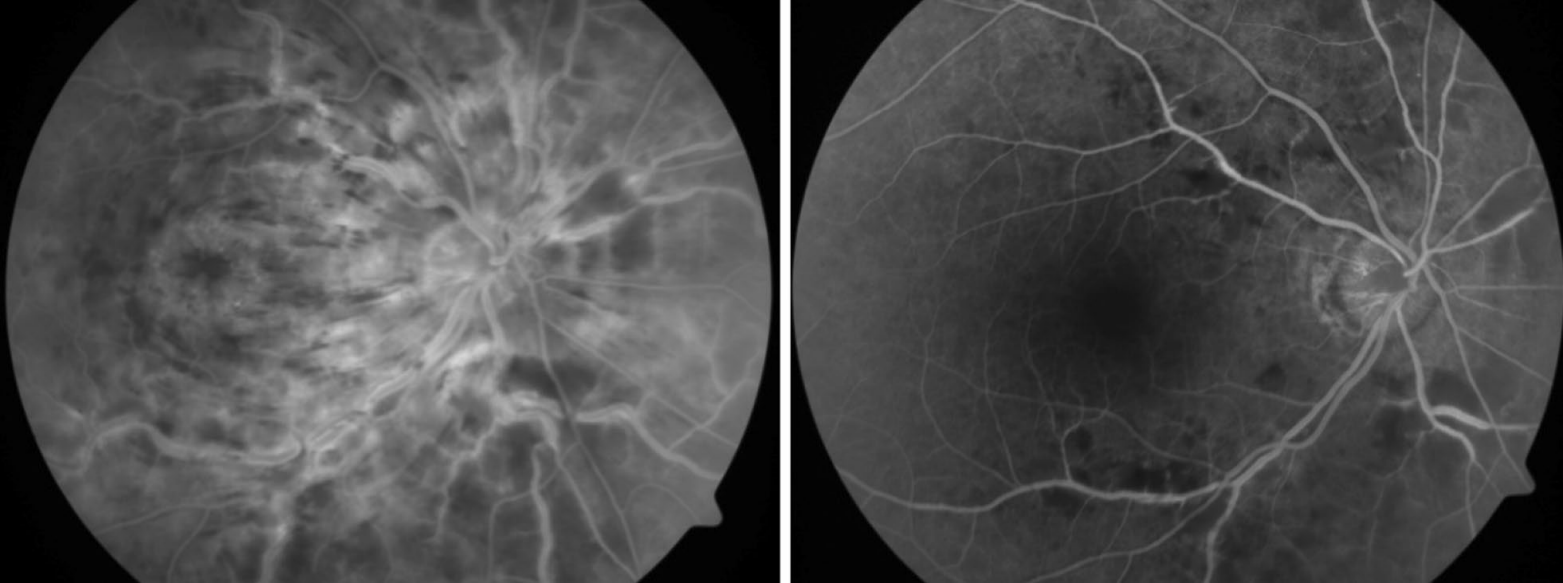
FFA pre and post Inj Ozurdex showing resolving macular oedema

**Fig. 3:**
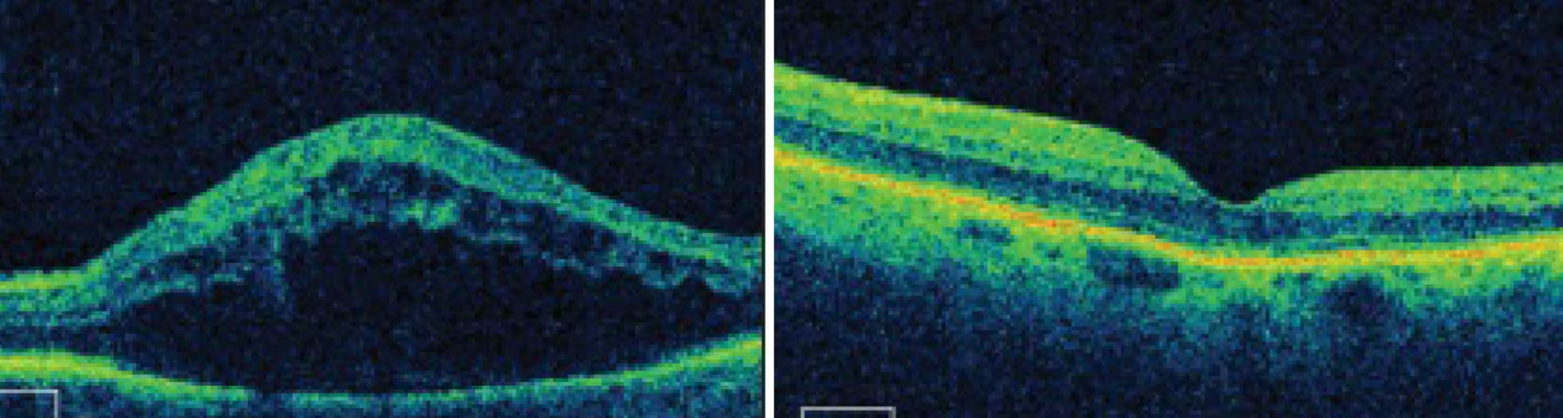
OCT macula pre and post Inj Ozurdex

**Fig. 4:**
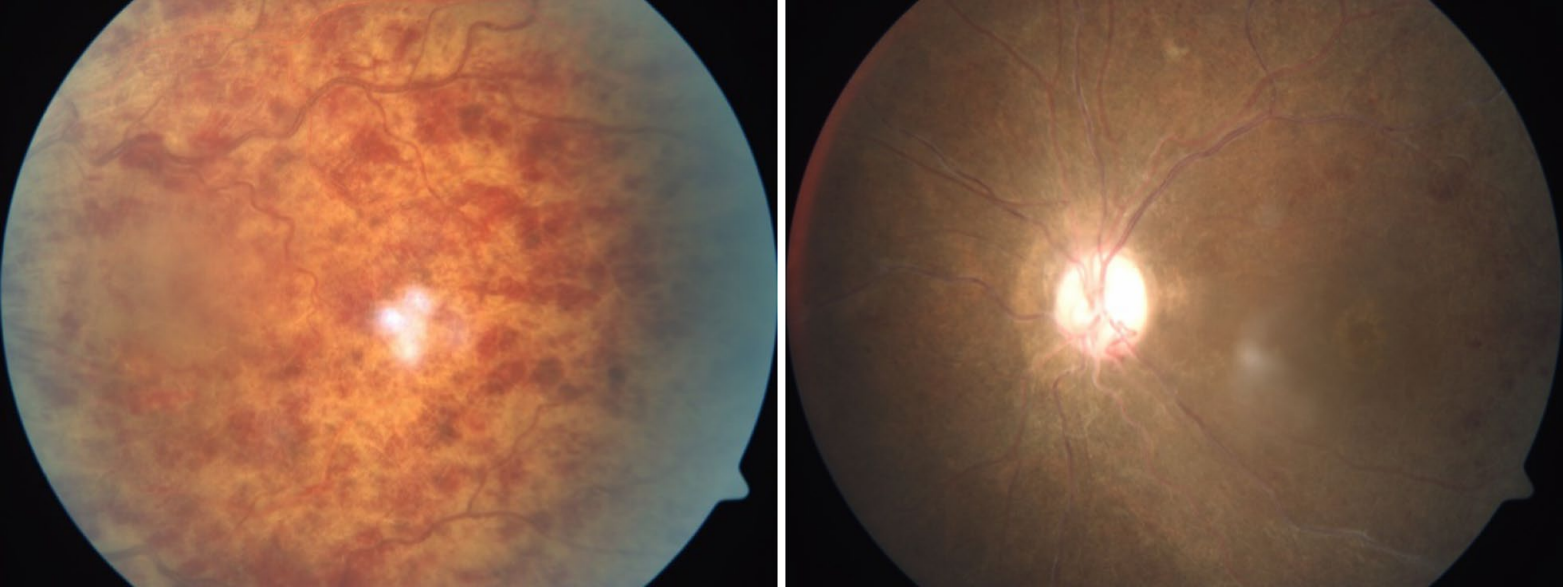
Fundus photo pre and post Inj triamcinolone

**Fig. 5:**
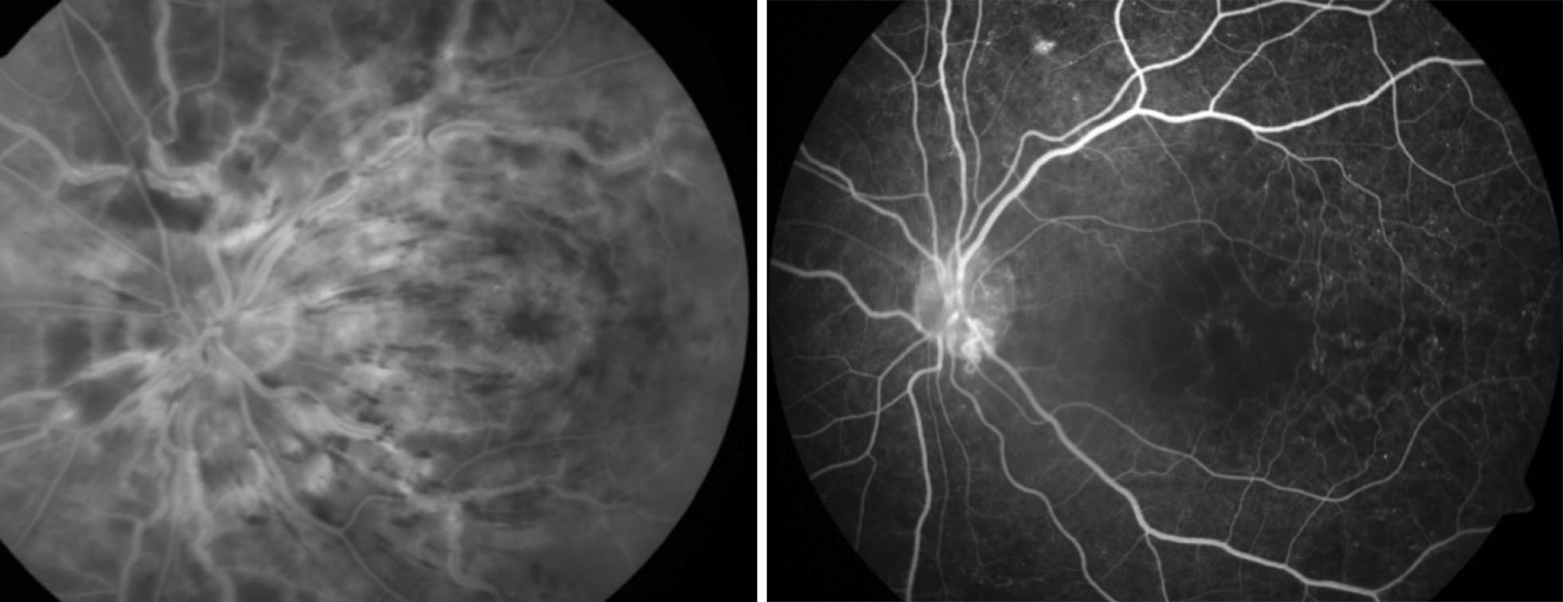
FFA pre and post Inj triamcinolone showing resolving macular oedema with capillary non perfusion areas and enlarged and distorted foveal avasular zone

**Fig. 6:**
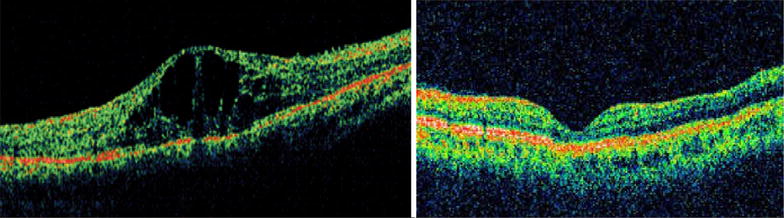
OCT macula pre and post Inj triamcinolone

Of the phakic eyes at baseline, 7 eyes (35%) in the IVTA group either had new onset lens opacity or progression of an existing opacity through month 6 as compared to 2 in the IVD group. In the IVTA group, 5 eyes (71.5%) of the 7 eyes with significant cataract progression, required cataract surgery at end of follow up period. Relative risk of cataract progression with IVTA is 3.5 times higher compared to IVD. No reports of infectious endophthalmitis were noted in either of the groups during the study period. Minor ocular adverse events related to injection procedure such as vitreous floaters and conjunctival haemorrhage occurred in similar number of eyes in both groups. Representative Fundus, FFA and OCT images before and after intervention with IVD is as seen in Figs. 1, 2 and 3 respectively and those after IVTA are as seen in Figs. 4, 5, and 6 respectively.

## Discussion

 Studies related to TCA done by Cekic et al. [10] reported average gain in VA in patients treated with TCA is 1.3 Snellen lines (range − 3 to 7) over the course of 10 months. Out of 24, 10 eyes gained ≥ 2 lines of VA, 3 eyes improved 1-line, 7 eyes remained the same, and 4 eyes worsened. There was no correlation between improvement in foveal thickness and corresponding visual gain. The SCORE [8] study group concluded that out of 271 patients, 27% participants in 4-mg TCA group achieved ≥ 15-letters improvement from the baseline. Jonas et al. [11] reported an improvement in VA by at-least 2 Snellen lines and 3 Snellen lines, respectively, in 8 (62%) eyes and 5 (38%) eyes treated with triamcinolone. VA measurements at 1 month (P = 0.038) and 3 months (P = 0.046) after the injection were significantly higher than the baseline values. Increase in VA was higher in the non-ischemic subgroup than the ischemic subgroup. Patel [12] et al. reported VA improvement ≥ 2 Snellen lines in eight (62%) eyes with mean VA improvement of 0.46 logmar with TCA intervention. The studies related to dexamethasone done by GENEVA [7] study group reported 22% participants in 0.7 mg dexamethasone group achieved ≥ 15-letters improvement from baseline. Joshi et al. [13] reported mean change in VA at 12 months with Ozurdex compared to baseline for CRVO was 11.5 ± 11.0 EDTRS letters, 30% of eyes gained ≥ 15 letters. Visual outcomes are similar to those previously seen with TCA in the SCORE study [8].

Our study shows that, likelihood of a gain in VA letter score of ≥ 15 at 6 months is similar in IVTA and IVD groups. At all-time points through 6 months, mean VA improvements are comparable between IVD and IVTA groups. At 6 months, mean improvement in BCVA was 0.43 and 0.49 logmar in IVD and IVTA groups, respectively and was not statistically significant between groups (Table 3). Within both groups there was significant improvement in VA at each follow up visit. In our study 8/20 subjects (40%) in both groups achieved ≥ 15 letters improvement from baseline till 24 weeks. This is in concurrence with other studies which have reported that steroids improve VA in patients of ME due to CRVO.

There was no statistical difference between groups in CMT at 6 months (Table 4). In both treatment arms 18/20 (90%) subjects achieved CMT of ≤ 250 microns at the end of 6 months period. In the GENEVA [7] study for dexamethasone implant 40% subjects achieved CMT < 250 microns at 6 months, while the SCORE study group reported 38% subjects with CMT of ≤ 250 microns at 6 months. Cekic O et al. [10] reported that in TCA treated patients the mean CMT decreased to 55% of preinjection values [(n = 23) 635 vs. 352 μm, respectively; P < 0.001]. Ip et al. [14] reported that mean baseline CMT before TCA injection was 590 µm and improved to 281 µm at 6-months in 13 patients. Patel et al. [12] reported that CMT decreased significantly by 259 μm (45% improvement) at best after treatment (SD 94 μm; *P *= 0.000017), with five eyes achieving a thickness ≤ 250 μm in 13 patients injected with triamcinolone.

In our study at 6 months, mean improvement in CMT was 323 and 322 µm in IVD and IVTA groups, respectively. In both treatment arms 18/20 (90%) subjects achieved CMT of ≤ 250 µm at 6 months and was statistically similar between groups. Comparatively greater proportion of patients achieved reduction in CMT. This may be attributed to selective inclusion of patients with shorter duration of ME of < 3 months yielding better prognosis than patients with longer duration ME. Within groups there was statistically significant reduction of CMT at each follow-up in both treatment arms (Table 5).

In regards to safety outcome for dexamethasone-implant, GENEVA [7] study reported that 15% study eyes showed IOP elevation of at-least 25 mmHg peaking at day 60 and normalising by day 180. While triamcinolone in SCORE [8] study reported 35% of study eyes with IOP rise and 8 subjects having IOP > 35 mmHg. Cekic et al. [10] reported that 9/18 patients without a history of glaucoma developed ocular hypertension and required glaucoma medication during post-TCA injection follow-up. Trabeculectomy was performed on 2 eyes with glaucoma. Ip et al. [14] reported that out of 13 patients on TCA, 1 patient experienced IOP rise that was controlled with 2 aqueous suppressants. Jonas et al. [11] reported that in the TCA group, IOP increased significantly (P = 0.018) from 14.4 ± 3.9 mmHg to a mean maximal value of 21.6 ± 9.2 mmHg (range 10–44 mmHg) and decreased (P = 0.012) towards the end of 10 month follow-up to 15.3 ± 5.1 mmHg (range 10–21 mmHg). Patel [12] reported an IOP rise in 8/13 (62%) eyes during follow-up. Maximum IOP < 30 mmHg was seen in 4 eyes, while < 40 mmHg was seen in 2 eyes and both these patients were adequately managed with topical medications. In total, 5 eyes (38%) required IOP lowering medication at the end of follow-up.

In our study, IVTA group required more intervention in terms of IOP lowering medications compared to IVD group. Twelve cases (60%) in IVTA group showed elevated IOP, of which 8 (66.6%) were managed with combination eye drops and difference was statistically significant from baseline at 6 months (Table 3). However, in IVD group IOP rise was manageable with single antiglaucoma medication and returned to baseline by 6 months (Table 3).

Endophthalmitis was not reported in either of these. Seven phakic eyes (35%) in IVTA group either had new onset lens opacity or progression of an existing opacity through month 6 as compared to 2 (10%) in the IVD group. In IVTA group, 5 of 7 (71.4%) with significant cataract progression required cataract surgery at 6 months while none required cataract surgery in IVD group.

Thus, our study demonstrates that dexamethasone 0.7 mg sustained release implant and triamcinolone-acetonide are effective in restoring vision in patients of early CRVO, with a high proportion (40%) gaining ≥ 3 lines. In terms of safety, drug induced IOP rise 35% lesser with dexamethasone implant compared to IVTA. With IVTA there is probability of 42% patients experiencing IOP rise > 35 mmHg and about 67% requiring combination anti-glaucoma medications and fail to normalise at 6 months. Cataract progression and propensity for cataract surgery is 25 and 100% lesser with dexamethasone implant at 6 months’ therapy. Our sample size is small, with longest follow up of 18 months, larger study with longer follow up is needed to establish these findings.

## Conclusion

 Intravitreal steroids are effective in managing macular edema of retinal vein occlusion, while newer formulation of sustained release dexamethasone implant is significantly safer than IVTA.
